# A discussion of statistical methods to characterise early growth and its impact on bone mineral content later in childhood

**DOI:** 10.1080/03014460.2019.1574896

**Published:** 2019-04-15

**Authors:** Sarah R. Crozier, William Johnson, Tim J. Cole, Corrie Macdonald-Wallis, Graciela Muniz-Terrera, Hazel M. Inskip, Kate Tilling

**Affiliations:** a MRC Lifecourse Epidemiology Unit, Southampton General Hospital, University of Southampton, Southampton, UK;; b School of Sport, Exercise and Health Sciences, Loughborough University, Loughborough, Leicestershire, UK;; c Population, Policy and Practice Programme, UCL Great Ormond Street Institute of Child Health, London, UK;; d MRC Integrative Epidemiology Unit, Oakfield House, University of Bristol, Bristol, UK;; e Department of Population Health Sciences, Oakfield House, Bristol, UK;; f Centre for Clinical Brain Sciences, University of Edinburgh, Edinburgh, UK

**Keywords:** Growth mixture models, lifecourse epidemiology, linear spline models, multilevel models, SITAR

## Abstract

**Background:** Many statistical methods are available to model longitudinal growth data and relate derived summary measures to later outcomes.

**Aim:** To apply and compare commonly used methods to a realistic scenario including pre- and postnatal data, missing data, and confounders.

**Subjects and methods:** Data were collected from 753 offspring in the Southampton Women’s Survey with measurements of bone mineral content (BMC) at age 6 years. Ultrasound measures included crown-rump length (11 weeks’ gestation) and femur length (19 and 34 weeks’ gestation); postnatally, infant length (birth, 6 and 12 months) and height (2 and 3 years) were measured. A residual growth model, two-stage multilevel linear spline model, joint multilevel linear spline model, SITAR and a growth mixture model were used to relate growth to 6-year BMC.

**Results:** Results from the residual growth, two-stage and joint multilevel linear spline models were most comparable: an increase in length at all ages was positively associated with BMC, the strongest association being with later growth. Both SITAR and the growth mixture model demonstrated that length was positively associated with BMC.

**Conclusions:** Similarities and differences in results from a variety of analytic strategies need to be understood in the context of each statistical methodology.

## Introduction

There is increasing interest in modelling longitudinal data and determining relationships with a future outcome. For example, a hypothesis that has been widely explored is the association between birth size, childhood growth and health outcomes in later life such as blood pressure. Various methods are available for examining such associations, but there has been limited work comparing their advantages and disadvantages; it is unclear whether the methods provide similar results, and there is little information describing which methods are most appropriate in particular situations.

Studies previously comparing methods include Tu et al. (2013), who analysed data on repeated weights from ages 1–19 years in relation to blood pressure at 19 years using methods including *z*-score plots, life course models, path analysis, conditional models, and latent variable models; there were no missing data, and no confounders were considered. De Stavola et al. (2006) compared conditional and joint models using two examples: maternal and grand-maternal influences on offspring size at birth, and the influence of childhood height on adult leg length. The examples had missing data, but did not adjust for confounders. Both papers concluded that more than one method of analysis would be useful to examine the robustness of conclusions to assumptions, and to answer different questions. Sayers et al. (2017) compared six methods that relate a linear trajectory of change to a later outcome, using simulated data, concluding that two-stage approaches result in biased unconditional associations. Johnson (2015) provides an overview of strategies available in modelling human growth, but does not apply these to one dataset in order to compare results. To date, no evaluation has examined prenatal growth or been based on an example including both missing data and confounders. Newer methods have recently been developed: the joint multilevel linear spline model and SITAR. Here we compare five methods, chosen because they are widely used to model longitudinal data and the relationship with a future outcome, but aren’t adversely affected by high levels of collinearity between measurements: a residual growth model, two-stage multilevel linear spline model, joint multilevel linear spline model, SITAR and a growth mixture model approach. The inclusion of newer methods and application of all methods to a realistically complex scenario including pre- and postnatal data, missing data and confounders, builds on previously published comparisons. The residual growth model and both multilevel linear spline models provide measures of growth in different time intervals, whereas the SITAR method enables researchers to model three parameters relating to the biological process of growth; the growth mixture model groups participants according to broad patterns of growth across the whole exposure period. All methods address the question of how these measures of growth are related to an outcome in later life.

Peak bone mass is achieved in the third to fourth decade of life and is a major determinant of osteoporotic fracture risk in later life (Hernandez et al. 2003); understanding factors that influence bone mineral accrual may, thus, inform novel approaches to fracture prevention. Our example relates linear growth (both prenatal and postnatal) to 6-year bone mineral content (BMC). The aim of the study is to demonstrate a variety of methods as illustrated by application to the example of childhood growth, and to provide some guidance as to when each method might be useful. We first describe the dataset and then the various models in turn. We then compare the results and identify strengths and weaknesses of each method for particular applications.

## The Southampton Women’s Survey

The Southampton Women’s Survey (SWS) is a prospective cohort study comprising 12,583 non-pregnant women living in Southampton, UK (Inskip et al. 2006). Women who became pregnant were followed up with ultrasound scans at 11, 19 and 34 weeks’ gestation, including measurements of crown–rump length (CRL) (at 11 weeks gestation) and femur length (FL) (at 19 and 34 weeks). Postnatally, crown–heel length was measured at birth, 6 and 12 months and height at 2 and 3 years. At 6 years of age a sub-set of children had whole body bone mass (omitting the head) measured by dual energy x-ray absorptiometry (DXA).

A total of 1852 women became pregnant and delivered a singleton term infant surviving the neonatal period, with no major congenital growth abnormalities, before the end of 2003. Of these, 1173 children born between February 2000 and December 2003 were visited at home at 6–7 years of age and 753 were subsequently willing to attend a clinic and have a whole body DXA scan and comprise the analysis sample. Of these, 432 participants had complete data for linear size at all ages; the median (range) number of linear size measures per participant was 8 (4–8).

Descriptive statistics for the 1852 infants born before the end of 2003, the sub-set of 753 with a DXA scan and the 432 with complete data for linear size are provided in Supplementary Table S1; broadly the groups are comparable, although there is a tendency for those having a DXA scan to be taller and slightly better educated than the baseline sample, and for those with complete linear size measurements to be again taller and better educated than those with a DXA scan.

Age was defined as years from predicted date of delivery (to adjust for gestational age at birth), e.g. 11 weeks’ gestation = −0.56 years. For the SITAR method, age was defined as years from birth because this analysis provides a data-driven developmental age adjustment. In these analyses sex is considered as a confounder, and adjustment for age at BMC measurement is included in order to account for age-related variability in BMCs. Descriptive statistics are given in Table 1.

**Table 1.** Descriptive statistics.

Characteristic	*n*	Value^*a*^
11 week crown-rump length (cm)	503	5.3 (0.8)
19 week femur length (cm)	712	3.1 (0.2)
34 week femur length (cm)	746	6.5 (0.3)
Birth supine length (cm)	737	50.1 (1.9)
6 month supine length (cm)	746	67.5 (2.5)
12 month supine length (cm)	735	75.9 (2.7)
2 year height (cm)	705	86.8 (3.1)
3 year height (cm)	719	96.1 (3.5)
Males, *n* (%)	753	393 (52%)
6 year BMC (kg)	753	0.54 (0.07)
Age at BMC (years)	753	6.7 (6.5–6.8)

a Percentage for categorical data, mean (SD) for continuous data except age at BMC, for which median (IQR) is presented.

**Figure 1.**
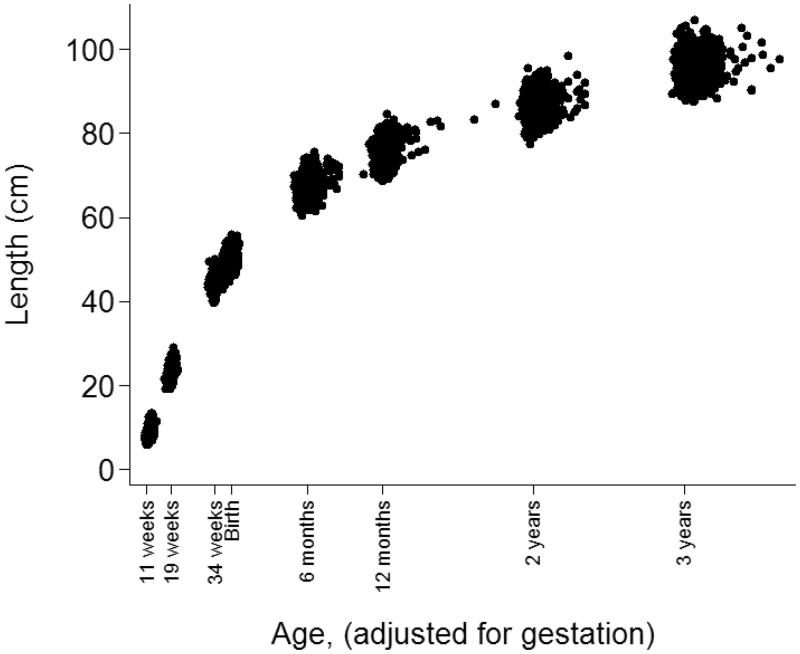
Length of SWS participants by age.

Measurements of length were not available from prenatal ultrasound scan measurements. However, length can be estimated from CRL and FL by assuming that they are proportional to total length. An appropriate multiplier was found by comparing the summary statistics for total length from foetal autopsies provided by Guihard-Costa et al. (2002) with those for CRL and FL in the SWS dataset. This suggested multipliers of 1.71, 7.66 and 6.91 to predict foetal length from CRL at 11 weeks and FL at 19 weeks and 34 weeks, respectively. Note also that there is a small discontinuity between supine length (measured at birth, 6 and 12 months) and height (measured at 2 and 3 years) that remains to be accounted for in the statistical methods. For convenience, scaled foetal length, supine length and height are henceforth referred to as *length*. Figure 1 shows how length varies by age in the sample. Age had a normal distribution at 11 weeks, 19 weeks and 34 weeks gestation and birth; the means (SD) were −0.54 (0.01), −0.39 (0.01), −0.11 (0.01) and 0.00 (0.02) years, respectively. Age had a skewed distribution at 6 and 12 months, 2 and 3 years; the medians (IQRs) were 0.51 (0.49–0.54), 1.02 (1.00–1.05), 2.04 (2.00–2.07) and 3.06 (3.02–3.12) years, respectively.

## Residual growth model

Sequential length measures taken in relatively close proximity are highly collinear, so regressing the outcome on all individual measurements leads to wide confidence intervals. Residual growth modelling, however, involves deriving independent measures of growth (Keijzer-Veen et al. 2005; Adair et al. 2009; Menezes et al. 2012).

Residual growth for child *j* is defined as the difference between observed length at time *p* (*length_pj_*) and predicted length at time *p* ${\hat{(\mathrm{length}}}_{\mathrm{pj}}$). ${\hat{\mathrm{length}}}_{\mathrm{pj}}$ is obtained by an ordinary least squares regression of *length_pj_* on all previous lengths:

$${\mathrm{length}}_{\mathrm{pj}}=\;\beta_{0}+\sum_{i=1}^{p-1}\beta_{i}{\mathrm{length}}_{\mathrm{ij}}+\;e_{j}$$

Residual growth (${\mathrm{length}}_{\mathrm{pj}}-\;{\hat{\mathrm{length}}}_{\mathrm{pj}})\;$is, therefore, the estimated residual error $\left({\hat{e}}_{j}\right)$ and is the growth in length relative to that predicted from all previous length measurements. The seven residual growth measurements are orthogonal to all preceding length measurements, and all preceding residual growth measurements and, thus, are independent of each other.

Although measurements were planned for defined ages, there was inevitable variability in the actual ages of measurement. Therefore, before the residual growth models were fitted, internal length *z*-scores were derived using the LMS method (Cole and Green 1992). This method provides smoothed centile curves to potentially skewed data, allowing *z*-scores to be calculated at exact ages; these *z*-scores were used in the growth residual model. The LMS method summarises the changing distribution of a measurement according to a covariate such as age by three curves representing the skewness (L), median (M) and coefficient of variation (S). Internal rather than external *z*-scores were chosen, since suitable external standards were not available for prenatal data. LMS *z*-scores were created using LMSchartmaker (Pan and Cole 2011) to fit growth curves for boys and girls separately.

**Figure 2.**
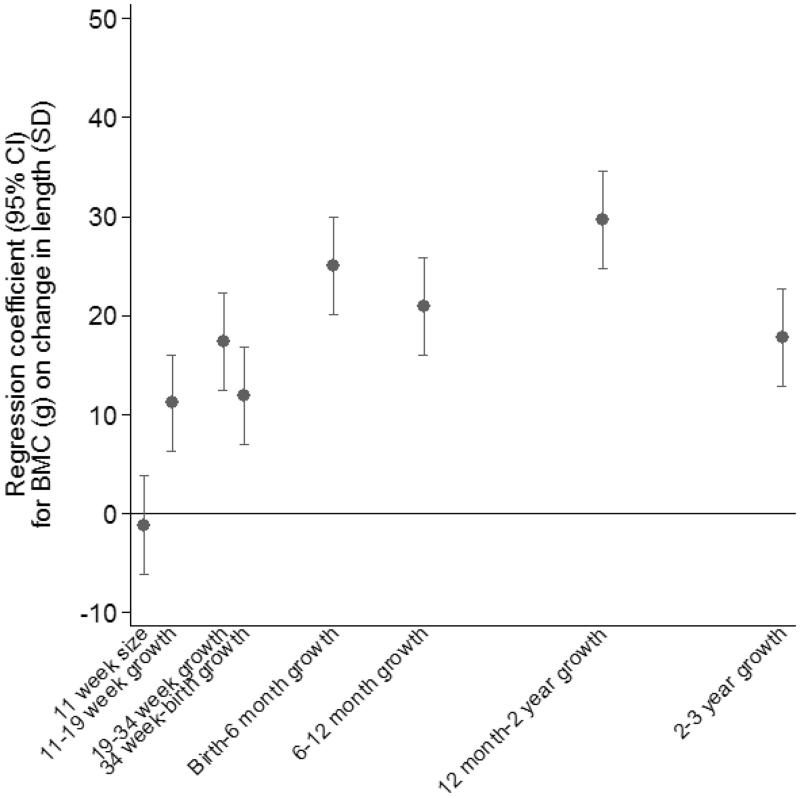
Residual growth modelling: conditional change in length as predictors of 6 year whole body BMC (g).

Residual growth measures were derived using all time points from 11 weeks’ gestation to 3 years and were scaled to have a mean of 0 and an SD of 1 so that the final coefficients would be comparable across time points. In a second stage, ordinary least squares regression was used to regress 6-year whole body BMC on size at the first time point and the residual growth measures, including sex and age at BMC as additional predictors. Four hundred and thirty-two participants who had complete data for length at all ages contributed to the analysis, which explained 55% of the variation in BMC. Figure 2 illustrates the results of the model. BMC was positively associated with faster residual growth between birth and 3 years, particularly between 12 months to 2 years.

## Two-stage multilevel linear spline model

A multilevel linear spline model (Tilling et al. 2011) was fitted to the repeated measurements of length. This model has two levels: measurement occasion (level 1) and within individual (level 2). It models the change in length with age as a piecewise linear growth trajectory between knot points where the slope changes and partitions the variation in the repeated measures of length into between-individual (level 2) and within-individual (level 1) variation. The exposure variable was age, and the outcome variable was length. Explanatory variables were included to indicate the type of measurement (i.e. CRL, FL, or supine length (reference; separate effects were not required for each of these) and height) and the level 1 variance was allowed to differ between CRL measures and non-CRL measures as this improved model fit. The knot points were positioned at the ages of measurement (except the extreme ages), since these were most informative about child’s length. Models were fitted including every combination of three- and four-knot linear splines (i.e. every possible set of knot positions); models with fewer than three knots were too simplistic for the shape of the curve and fitted the data poorly, while models with more than four knots failed to converge. The selected model had a low AIC and also minimised the mean absolute differences between the observed and predicted values across measurement occasions. It had knots at 34 weeks’ gestation, birth, 6 and 12 months of age, with a baseline at 11 weeks’ gestation, thus estimating average size at 11 weeks’ gestation (*β*_0_) and changes between 11 and 34 weeks gestation (*β*_1_), 34 weeks gestation and birth (*β*_2_), birth and 6 months (*β*_3_), 6 and 12 months (*β*_4_) and 12 months and 3 years (*β*_5_). Individual-level random effects were allowed for all these estimates, meaning that each individual is allowed to have their own size at 11 weeks gestation and changes between time points. The model estimates the variances and covariances of these individual deviations from the average pattern of growth. For example, the between-individual variance in size at 11 weeks gestation is given by $\sigma_{u0}^{2}$ below. The *i*th measurement for the *j*th individual is given by:

$$l\mathrm{engt}h_{\mathrm{ij}}=\beta_{0}+(\beta_{1}+u_{1j})\mathrm{spline}1{\left(11-34w\mathrm{eeks}\right)}_{\mathrm{ij}}+(\beta_{2}+u_{2j})\mathrm{spline}2{\left(34w\mathrm{eeks}-\mathrm{birth}\right)}_{\mathrm{ij}}+(\beta_{3}+u_{3j})\mathrm{spline}3{\left(\mathrm{birth}-6m\mathrm{onths}\right)}_{\mathrm{ij}}+(\beta_{4}+u_{4j})\mathrm{spline}4{\left(6-12m\mathrm{onths}\right)}_{\mathrm{ij}}+(\beta_{5}+u_{5j})\mathrm{spline}5{\left(12m\mathrm{onths}-3y\mathrm{ears}\right)}_{\mathrm{ij}}+\mathrm{CR}L_{\mathrm{ij}}+\mathrm{heigh}t_{\mathrm{ij}}+u_{0j}+\mathrm{CR}L_{\mathrm{ij}}\times \varepsilon_{\mathrm{CR}L_{\mathrm{ij}}}+(1-\mathrm{CR}L_{\mathrm{ij}})\times \varepsilon_{\mathrm{non}-\mathrm{CR}L_{\mathrm{ij}}}$$

where

$$\left[\begin{array}{c} u_{0j}\\ u_{1j}\\ u_{2j}\\ u_{3j}\\ u_{4j}\\ u_{5j} \end{array}\right]∼\mathrm{Normal}\left(\left[\begin{array}{c} 0\\ 0\\ 0\\ 0\\ 0\\ 0 \end{array}\right],\left[\begin{array}{cccccc} \sigma_{u0}^{2} & & & & & \\ \sigma_{u01} & \sigma_{u1}^{2} & & & & \\ \sigma_{u02} & \sigma_{u12} & \sigma_{u2}^{2} & & & \\ \sigma_{u03} & \sigma_{u13} & \sigma_{u23} & \sigma_{u3}^{2} & & \\ \sigma_{u04} & \sigma_{u14} & \sigma_{u24} & \sigma_{u34} & \sigma_{u4}^{2} & \\ \sigma_{u05} & \sigma_{u15} & \sigma_{u25} & \sigma_{u35} & \sigma_{u45} & \sigma_{u5}^{2} \end{array}\right]\right)$$

and

$$\left[\begin{array}{c} \varepsilon_{\mathrm{CR}L_{\mathrm{ij}}}\\ \varepsilon_{\mathrm{non}-\mathrm{CR}L_{\mathrm{ij}}} \end{array}\right]∼\mathrm{Normal}\left(\left[\begin{array}{c} 0\\ 0 \end{array}\right],\left[\begin{array}{cc} \sigma_{\varepsilon 1}^{2} & \\ 0 & \sigma_{\varepsilon 2}^{2} \end{array}\right]\right)$$

In the second stage, BMC was regressed on each of the standardised individual-level random effects from the multilevel model in turn, adjusting for sex, age at BMC measurement and individual-level random effects relating to earlier age intervals and length at 11 weeks. For example, to estimate the association of growth between birth and 6 months conditional on prior growth with BMC, the regression equation is given by:

$$\mathrm{BM}C_{j}=\alpha_{0}+\alpha_{1}sex_{j}+\alpha_{2}B\mathrm{MCag}e_{j}+\alpha_{3}{\hat{u}}_{0j}+\alpha_{4}{\hat{u}}_{1j}+\alpha_{5}{\hat{u}}_{2j}+\alpha_{6}{\hat{u}}_{3j}$$

where ${\hat{u}}_{0j},$ ${\hat{u}}_{1j},$ ${\hat{u}}_{2j}$ and ${\hat{u}}_{3j}$ are the standardised best linear unbiased predictors of the random effects from the multilevel model and $\alpha_{6}$is the regression coefficient of interest.

Bootstrapping (by cluster with replacement, using 500 replications) was used to derive confidence intervals for the coefficients in order to account for the uncertainty in estimating the growth parameters using the multilevel model. For each bootstrap sample, the multilevel model was fitted, random effects estimated and then BMC regressed on the standardised random effects. The 2.5^th^ and 97.5^th^ percentiles were used as the confidence intervals for each parameter.

**Figure 3.**
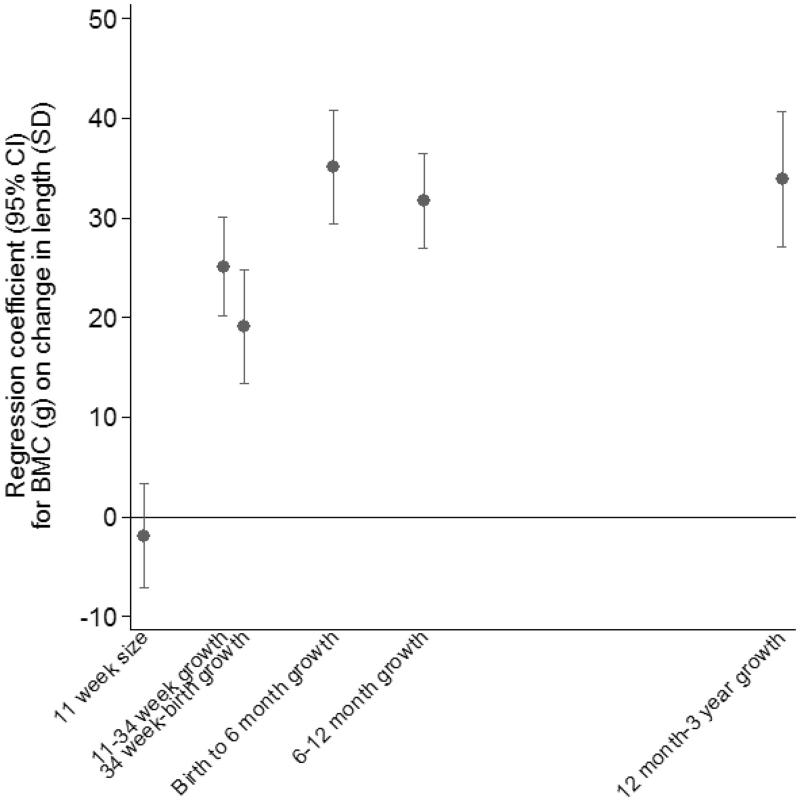
Two stage multilevel linear spline: conditional change in length as predictors of 6 year whole body BMC (g).

Figure 3 illustrates the regression coefficients from the second stage regression model. The model was fitted on all 753 participants and explained 53% of the variance in BMC. After adjustment for growth in earlier periods, growth in all periods up to 3 years was strongly positively associated with BMC. The greatest difference in BMC per SD of growth was seen for growth between birth and 6 months.

## Joint multilevel linear spline model

A bivariate multilevel linear spline model (Macdonald-Wallis et al. 2012) was fitted to the repeated measurements of length and to BMC, all as outcomes. The model had three levels: measurement (length or BMC, level 0) within measurement occasion (level 1) and within individual (level 2). The exposure variable for the multilevel model for length was age, with knot points at 34 weeks gestation, birth, 6 and 12 months of age, so that the results were comparable with the two-stage multilevel linear spline model. The exposure variables for the BMC outcome were sex and age at BMC measurement (centred at 6.5 years). The model, therefore, included the same six individual-level random effects as in the two-stage multilevel linear spline model (above) plus an individual-level random effect for BMC. This allows individuals to have growth that varies around a population average, BMC that varies around a population average, and for the individual growth and BMC to be related. These seven random effects were used to derive estimates of the coefficients for the regression of BMC on each growth parameter, adjusted for previous growth (Tilling et al. 2001; Goldstein et al. 2002; Macdonald-Wallis et al. 2012). The formula for the *i* ^th^ measurement occasion and the *j* ^th^ individual is given by:

$$l\mathrm{engt}h_{\mathrm{ij}}=\beta_{0}+(\beta_{1}+u_{1j})\mathrm{spline}1{\left(11-34w\mathrm{eeks}\right)}_{\mathrm{ij}}+(\beta_{2}+u_{2j})\mathrm{spline}2{\left(34w\mathrm{eeks}-\mathrm{birth}\right)}_{\mathrm{ij}}+(\beta_{3}+u_{3j})\mathrm{spline}3{\left(\mathrm{birth}-6m\mathrm{onths}\right)}_{\mathrm{ij}}+(\beta_{4}+u_{4j})\mathrm{spline}4{\left(6-12m\mathrm{onths}\right)}_{\mathrm{ij}}+(\beta_{5}+u_{5j})\mathrm{spline}5{\left(12m\mathrm{onths}-3y\mathrm{ears}\right)}_{\mathrm{ij}}+\mathrm{CR}L_{\mathrm{ij}}+\mathrm{heigh}t_{\mathrm{ij}}+u_{0j}+\varepsilon_{\mathrm{CR}L_{\mathrm{ij}}}+\varepsilon_{\mathrm{non}-\mathrm{CR}L_{\mathrm{ij}}}$$

$$\mathrm{BM}C_{j}=\beta_{6}+\beta_{7}sex_{j}+\beta_{8}B\mathrm{MCag}e_{j}+u_{6j}$$

where

$$\left[\begin{array}{c} u_{0j}\\ u_{1j}\\ u_{2j}\\ u_{3j}\\ u_{4j}\\ u_{5j}\\ u_{6j} \end{array}\right]∼\mathrm{Normal}\left(\left[\begin{array}{c} 0\\ 0\\ 0\\ 0\\ 0\\ 0\\ 0 \end{array}\right],\left[\begin{array}{ccccccc} \sigma_{u0}^{2} & & & & & & \\ \sigma_{u01} & \sigma_{u1}^{2} & & & & & \\ \sigma_{u02} & \sigma_{u12} & \sigma_{u2}^{2} & & & & \\ \sigma_{u03} & \sigma_{u13} & \sigma_{u23} & \sigma_{u3}^{2} & & & \\ \sigma_{u04} & \sigma_{u14} & \sigma_{u24} & \sigma_{u34} & \sigma_{u4}^{2} & & \\ \sigma_{u05} & \sigma_{u15} & \sigma_{u25} & \sigma_{u35} & \sigma_{u45} & \sigma_{u5}^{2} & \\ \sigma_{u06} & \sigma_{u16} & \sigma_{u26} & \sigma_{u36} & \sigma_{u46} & \sigma_{u56} & \sigma_{u6}^{2} \end{array}\right]\right)$$

and

$$\left[\begin{array}{c} \varepsilon_{\mathrm{CR}L_{\mathrm{ij}}}\\ \varepsilon_{\mathrm{non}-\mathrm{CR}L_{\mathrm{ij}}} \end{array}\right]∼\mathrm{Normal}\left(\left[\begin{array}{c} 0\\ 0 \end{array}\right],\left[\begin{array}{cc} \sigma_{\varepsilon 1}^{2} & \\ 0 & \sigma_{\varepsilon 2}^{2} \end{array}\right]\right).$$

**Figure 4.**
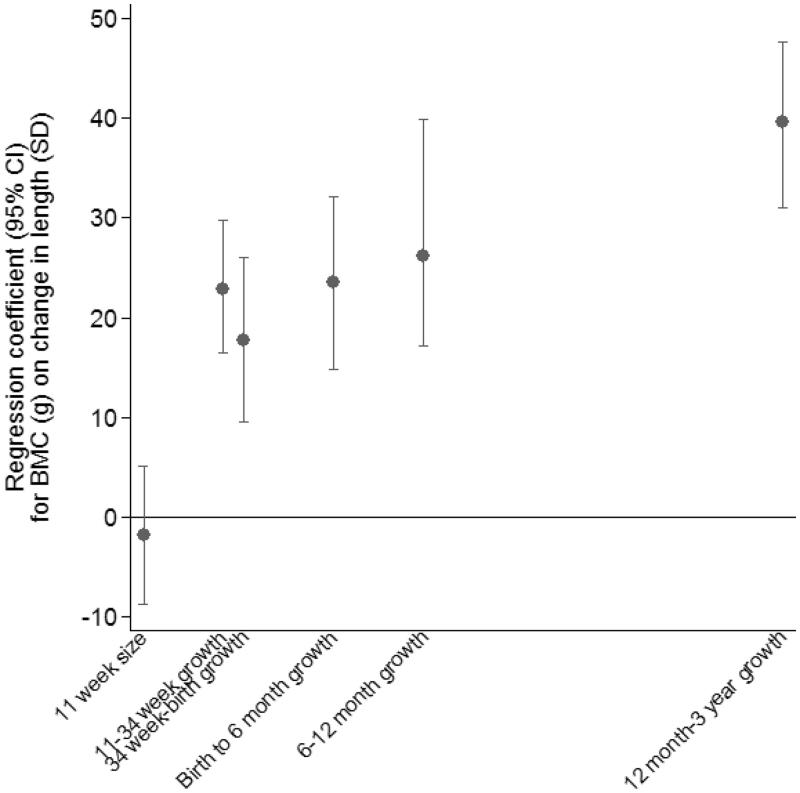
Joint multilevel linear spline: conditional change in length as predictors of 6 year whole body BMC (g).

The Stata command reffadjust (Palmer et al. 2014) was used to obtain confidence intervals for each coefficient. Figure 4 illustrates the regression coefficients from the model explaining 53% of the variance in BMC. After adjustment for growth in earlier periods, growth in all periods up to 3 years was strongly positively associated with BMC. The greatest difference in BMC per SD of growth was seen for growth between 12 months and 3 years.

## SITAR

SITAR (SuperImposition by Translation And Rotation) is a shape-invariant mixed effects growth model (Lindstrom 1995; Beath 2007; Cole et al. 2010) that models both the measurement scale and the age scale of the growth curve. The mean growth curve is fitted as a cubic spline and, in addition, the intercept on the measurement scale includes a subject-specific random effect, while on the time scale both the intercept and slope include subject-specific random effects. This means the model is ‘shape-invariant’—a single mean curve is estimated, but it is modified by the inclusion of the random effects to match the observed growth curves for individuals.

The three random effects reflect simple transformations of the mean curve. SITAR size is an up–down shift of the curve (random intercept on the measurement scale), SITAR timing is a left–right shift of the curve (random intercept on the age scale), and SITAR intensity is a shrinking–stretching of the age scale (random slope on the age scale). If the model fits well, adjusting the individual curves for their random effects superimposes them on the mean curve, leading to the name SITAR.

The formula for the SITAR model is

$${\mathrm{length}}_{\mathrm{ij}}=\alpha_{j}+h\left(e^{\gamma_{j}}\left(t_{\mathrm{ij}}-\beta_{j}\right)\right)+\varepsilon_{\mathrm{ij}}$$

where the length*_ij_* are measurements at ages *t_ij_*, with *i* indexing the occasion and *j* the subject; *h*(.) is a function in transformed age defining the mean spline curve; *α_j_*, *β_j_* and *γ_j_* are subject-specific random effects for size, timing and intensity, respectively, and the *ε_ij_* are normally distributed residuals. Fixed effects for *α*, *β* and *γ* are also included, to ensure the mean random effects are zero. Note that *γ* is exponentiated to provide a multiplier centred on one. The mean curve is fitted as a natural cubic B-spline, which is a cubic spline with particular properties where the number and placement of knots is chosen to minimise the Bayesian Information Criterion. The spline curve regression coefficients are estimated as fixed effects simultaneously with the other fixed and random effects. The model is fitted in R using the nlme package and the author’s sitar package.

The relationship between growth and later outcome can be estimated in at least three ways. The most obvious approach is to first fit the SITAR model and then as a second stage regress the outcome on the triplets of subject-specific SITAR random effects, analogous to the two-stage multilevel linear spline model described above. An alternative, though counter-intuitive approach is to include the outcome in the SITAR analysis as a fixed effect subject covariate for each of *SITAR size*, *SITAR timing* and *SITAR intensity*. In other words, the random effects for each subject are adjusted for the subject’s later outcome. This analysis effectively reverses time, by seeing whether the outcome (i.e. BMC here) ‘explains’ the earlier pattern of growth, hence testing for the existence or not of an association between growth and later outcome. A third approach is to fit the full bivariate spline model analogous to that in the previous section.

The last approach would be best, but was challenging analytically due to modelling the SITAR random effects on the age scale. Of the other approaches, the first assumes that the random effects are estimated without error, so that their standard errors need to be bootstrapped. However, in practice this led to unstable models with inconsistent results and the approach was dropped. The second approach assumes (incorrectly) that the outcome is measured without error; however, it was preferred as it correctly handles the uncertainty in the random effects and in practice it also gave more consistent results.

The SITAR model was fitted using $\sqrt{l\mathrm{ength}}$ and $\sqrt{a\mathrm{ge}+0.75}$ (after testing untransformed and log transformed alternatives), where the age offset of 0.75 years reflected the prenatal period. The model included all 753 participants in the dataset, using spline curves with knots at the three age quartiles (i.e. four degrees of freedom), and it explained 82% of the variance in length; this should be compared with values of around 95% usually seen for SITAR applied to weight in infancy or 99% for height in puberty.

**Figure 5.**
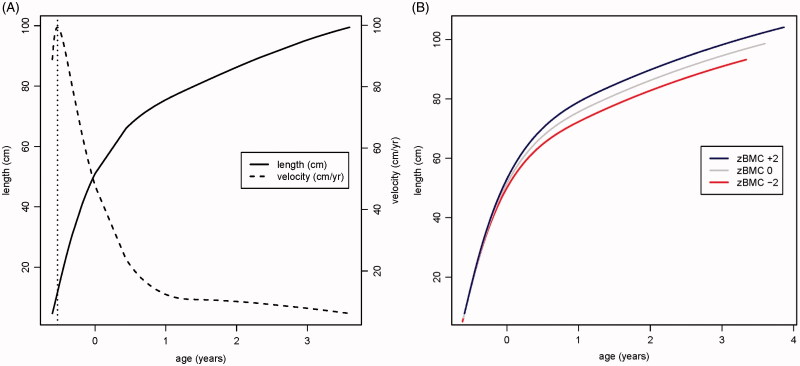
(A) Mean distance and velocity curves for length back-transformed from the square root scale. Age at peak velocity is marked. (B) SITAR-predicted length growth curves corresponding to BMC *z*-scores of −2, 0 and +2, respectively.

Figure 5A shows the fitted mean curves for back-transformed length and its first derivative (length velocity); the peak velocity was at 16 weeks’ gestation. In the analyses including age-adjusted BMC as a covariate to explain the three SITAR random effects, the most important covariate was *SITAR size* (*t* = +14.3), while *SITAR timing* (*t* = +5.3) and *SITAR intensity* (*t* = −3.8) were also highly significant. *SITAR size* is effectively a measure of mean length from 11 weeks gestation to 3 years; a child who was relatively long was likely to have greater BMC at age 6 years. *SITAR timing* marks the age when individuals are growing fastest, and this occurred early in pregnancy (Figure 5A). Thus, the age when a child is growing fastest is primarily a marker of early growth in this dataset. A later peak predicts higher BMC. *SITAR intensity* measures upward centile crossing in length across the age range and was negatively associated with BMC.

The three SITAR parameters were highly correlated (*size*-*timing r* = 0.76, *timing*-*intensity r* = −0.98, *size*-*intensity r* = −0.76). Figure 5B shows the predicted length growth curves corresponding to BMC *z*-scores of −2, 0 and +2, respectively, demonstrating the combined effects of the three BMC SITAR parameters on each curve. Taller infants who grew more quickly had a higher BMC (blue curve), while shorter infants who grew more slowly had a lower BMC (red curve).

## Growth mixture model

Growth mixture modelling (Muthén 2001, 2004) is used to identify distinct groups of individuals who share similar average growth curves by combining a latent curve model (to estimate the average growth curves) with a categorical latent class variable.

**Figure 6.**
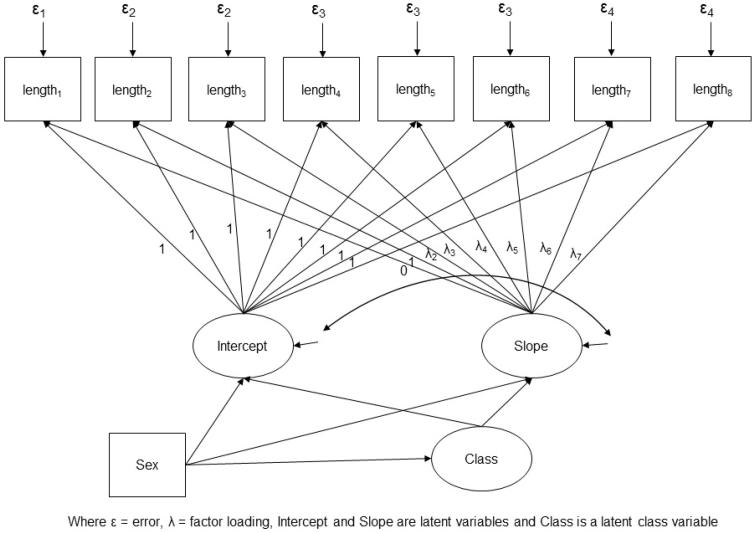
Growth mixture model.

A single growth model was fitted to estimate class membership and relate this to BMC. We fitted a non-linear spline that best fitted the data between any two time points (for the slope factor the first loading for length at 11 weeks was 0, and the second loading for length at 19 weeks was 1; the rest were freely estimated) (Bollen and Curran 2006). Variance and covariance of the latent growth variables (i.e. intercept and slope) were each constrained to be equal across classes. In addition, residual variances of the CRL, FL, supine length and height measurements were each constrained to be equal across classes. The latent growth variables and the categorical latent class variable were adjusted for sex. BMC was regressed on sex and age at BMC and the categorical latent class variable was related to BMC by estimating the sex- and age-adjusted mean BMC within each class. The model is described in Figure 6.

Mplus was used to estimate a series of growth mixture models with increasing numbers of classes (starting at two). The best model was chosen based on (1) overall fit according to the BIC, (2) quality of classification judged by the entropy statistic and (3) interpretability of the classes.

**Figure 7.**
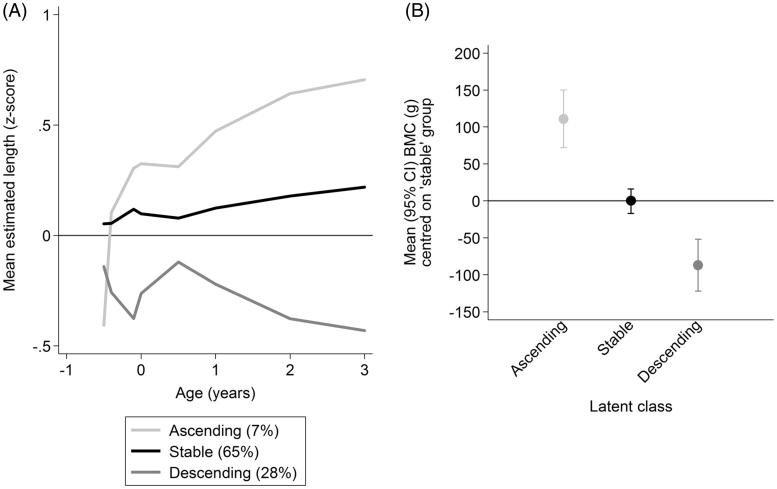
(A) Growth mixture model: average estimated length LMS *z*-scores by class and (B) Growth Mixture Model: Differences in BMC (g) from ‘stable’ group.

Three latent classes were identified, but with only reasonable separation (entropy = 0.69). For each participant at each time point sex-specific LMS *z*-scores were calculated for the estimated length measurements. Figure 7A characterises the classes by plotting average internal *z*-scores in each class. The largest class (65% of the sample) had a stable growth pattern, the second largest class (28%) had a descending pattern and a third (7%) described an ascending pattern. Differences in BMC in the ‘descending’ and ‘ascending’ classes compared with the ‘stable’ class are illustrated in Figure 7B; all pairwise comparisons were significant.

## Discussion

The findings from residual growth modelling and both multilevel linear spline models are the most straightforward to compare and showed that all measures of growth were positively associated with BMC. Postnatal growth was generally more closely related to BMC at age 6 years than earlier growth, although there were some differences in the exact associations, such as the period of growth most strongly related to the outcome. Results from the growth mixture model are less directly comparable, but again it was the fastest-growing children (in the ascending class) who had highest BMC. According to the SITAR analysis, taller infants who grew more quickly had a greater bone mass at age 6 years. The fact that there is broad agreement between these approaches, despite different underlying assumptions, gives us confidence in the conclusion that more rapidly-growing children have higher BMC at 6 years, and that this is robust to the modelling approach used. The percentage variance in BMC explained by the residual growth model (55%) was very similar to that from the multilevel linear spline models (both 53%); these percentages could not be determined for the SITAR and growth mixture models.

The residual growth modelling approach does not model the trajectory of growth, so gives no information about growth patterns. All the other methods describe growth and then relate summaries of the pattern of growth to the outcome. The linear spline models assumed a (unrealistic) piecewise linear pattern, whereas both SITAR and the growth mixture models allowed a more plausible non-linear trajectory. However, the associations estimated using linear spline multilevel models are easier to interpret than those estimated using smoother curve shapes.

Residual growth modelling has the advantage that growth between all time points is modelled, thus allowing the influence of all periods to be considered, although potentially also being susceptible to the issue of multiple comparisons. It is also relatively easy to understand and communicate to non-statisticians and is straightforward to implement in standard statistical software. The model can be extended to incorporate other predictors and to consider interactions. A limitation is that only participants with data at all time points can be analysed, resulting in loss of power and potential bias. Here 432 children were included, compared with 753 in all other models. The method is also only feasible for studies with data collected at more or less fixed ages, as in the SWS. Because of measurement error, effect sizes are likely to be biased towards the null, and uncertainty in the estimates of growth are not taken into account in the confidence intervals for the associations with BMC, thus standard errors may be under-estimated (Sayers et al. 2017).

Multilevel models (including the linear spline models and SITAR) are suitable for data measured at varying ages between participants, and where individuals have differing numbers of measurements. They can also allow for different measurement types (CRL, FL, supine length and height). A key decision in the linear spline models was the parameterisation of the trajectory—the linear spline provided ease of interpretation, but other possibilities include polynomials, non-linear splines and non-linear models (e.g. SITAR). With spline models further choices are the number and placement of knots (Tilling et al. 2014). All the multilevel models assume normality of random effects and residuals.

The two-stage multilevel linear spline model is relatively easy to implement and interpret. The initial stage of the analysis can be carried out by an experienced statistician; it is then simple for less experienced researchers to perform the second stage of the analysis. However, the correlations between the individual-level random effects were high (up to 0.8 for 6–12 months and 12 months–3 years), potentially leading to wider confidence intervals in the final model. Others have shown (Sayers et al. 2017) that this method is biased for all coefficients except for the final growth period (where all random effects are included), and that standard errors may be under-estimated as they do not incorporate the uncertainty in the estimation of the random effects.

If correctly specified, the joint multilevel linear spline model gives unbiased estimates of the relationship between BMC and growth, and correct standard errors. This leads to more accurate confidence intervals, which are notably wider (Figure 4) than those for the residual growth modelling (Figure 2) and the two-stage multilevel linear spline model (Figure 3). Disadvantages are that the method is only appropriate when the health outcome is continuous, and it is relatively complex to implement.

There are three main assumptions underlying SITAR: developmental age linearly related to chronological age, normality of residuals and multivariate normality of random effects. The first assumption may well be invalid in this example, since the time period includes both foetal and infant life when growth patterns can differ. Infants born earlier tend to grow faster postnatally, i.e. catch up, and, hence, are developmentally advanced. But their being born early may well be linked to reduced foetal growth, implying delayed prenatal development. So at birth they switch from being delayed to being advanced, and this contravenes the SITAR linearity assumption. That said, the sample here was term-born, which restricted the range of gestational ages to 37–42 weeks, but even so it reflects a wide spectrum of maturity.

SITAR provides a cubic spline mean curve and summary of growth in three parameters: size, timing and intensity of growth; this small number is attractive if the models fit well. The model operates on both the measurement and age scales, accounting for differences in developmental age. A disadvantage of SITAR is that it requires a non-linear mean curve or else the size and timing random effects become confounded. Also, a joint model of growth and a health outcome cannot (within currently available software) be fitted, meaning that the relationship of interest cannot be directly estimated. Instead, growth curves were related to later BMC (Figure 5B).

The growth mixture model classifies participants according to their pattern of growth. This has the strength of ease of interpretation, as long as growth in individual groups can be clearly described. Independent variables can be included to investigate systematic differences in average growth curves and estimates can be allowed to vary across the classes. These models are particularly appropriate if it is not tenable that all participants have the same underlying pattern of growth. Three groups were identified here, though inevitably some children did not neatly fit into any one group and were allocated to the group they most closely resembled. This model did identify a class of children in an ascending trajectory who had higher BMC at age 6 years; however, in theory, there may be patterns of growth that relate to the outcome which are not revealed by this method. Growth mixture models are computationally challenging, thus limiting models to low order polynomials. Also, model selection involves subjective judgements, the models may identify spurious classes and interpretation of the latent classes is not always straightforward. A further disadvantage is that, as it is the association between the latent class and BMC that is being estimated, relationships between BMC and growth during different age periods cannot easily be identified.

A key requirement of all analysis methods was for considerable pre-processing of the data: if the research question is about growth over time, then measures at all time points need to have the same meaning. Here, we achieved this by use of multipliers—although this still leaves the possibility of mean changes between measurement types (e.g. from supine length to height), and differing measurement error between methods or across ages. Combining prenatal and postnatal measurements involves making assumptions, and the development of multipliers using a sample of foetal autopsies may involve bias due to possible pathological growth, but measurements both before and after birth must be used if hypotheses regarding mismatch of the pre- and postnatal environment (Godfrey et al. 2007) are to be explored.

Residual growth models and linear spline models enable a researcher to discover how growth during different periods relates to an outcome. The results of these models were broadly consistent, such that growth, particularly at later ages, was positively associated with BMC. The choice between these methods in another context would depend upon: the research question of interest; whether measurements were made at fixed ages for all cohort members; the amount and structure of missing data; the statistical software available and the sensitive periods of interest. Bias in both residual growth models and the two-stage multilevel approach will increase with increasing measurement error.

The linear spline models require the assumption of piecewise linear growth, or at least that there are periods with approximately linear growth. If this assumption is untenable, other parameterisations could be explored. SITAR allows researchers to discover how three parameters relating to the biological process of growth are associated with the outcome of interest, allowing for a non-linear growth trajectory. The growth mixture models used here also allowed a non-linear growth trajectory, and this method of freely estimating the trajectory could be used in a non-mixture multilevel model. We recommend that, if a linear spline model is used as the main analysis model, a non-linear method should be used as a sensitivity analysis. Similarly, if a method assuming one pattern of growth is used as the main analysis model, a mixture model would be a useful addition to verify robustness of conclusions to this assumption.

The challenge for future researchers is to decide on the most appropriate methods of analysis to employ, the most important issue being the question under consideration. The choice of method will further depend on the nature of the measurements, the requirements for communication of the findings, the software available and whether assumptions inherent in the particular approach are met. It should also be noted that different methods achieve varying levels of data reduction. We have not attempted to perform a comprehensive comparison of all statistical methods to characterise growth, and the comparison made here does not point to one method that should be used in preference to others, but it has highlighted some of the issues that need to be considered. Table 2 provides brief guidance about when each approach might be useful. Since each method has its limitations, we agree with previous authors (De Stavola et al. 2006; Tu et al. 2013) that different methods of analysis can be considered complementary and more than one approach may be helpful to describe associations between longitudinal exposure data and a distal outcome. The approaches used should be carefully chosen to make different assumptions and a qualitative judgement made about agreement between different models.

**Table 2.** Guidelines for choice of statistical method to characterise growth.

Question	Data characteristics	Approach
How does growth relate to a later outcome?	Measures taken at same time for everyone. Little/no missing data. Fairly small number of measures. Outcome can be continuous or categorical.	*Residual growth model* Advantages: simple to implement and interpret. Disadvantages: does not describe pattern of growth; can only relate outcome to growth at ages measured; difficult to use where there are large amounts of missing data.
What is the pattern of growth, how does it vary between individuals, how does it relate to a later outcome?	Measures do not need to be at same times for everyone, nor does everyone need to have the same number of measures. Measures not too close together. Outcome can be continuous or categorical.	*Two-stage multilevel linear spline model* Advantages: fairly simple to interpret, pattern of growth modelled in an intuitive way. Can be moderately simple to implement. Disadvantages: Assumes periods of linear growth; biased associations with outcome unless all random effects included in model; autocorrelation may be a problem (if measures close together). If pattern of growth complex, model convergence may be problematic.
What is the pattern of growth, how does it vary between individuals, how does it relate to a later outcome?	As above, but with continuous (Normally distributed) outcome.	*Joint multilevel linear spline model* Advantages: Interpretable results for both pattern of growth and association with outcome. Unbiased (providing model correctly specified). Disadvantages: Can be complex to fit, and model convergence may be problematic.
How does growth vary with chronological and developmental age? How does this relate to a later outcome?	As for two-stage multilevel linear spline models.	*SITAR* Advantages: Biological interpretation to the association between growth and later outcome. Fewer parameters than linear spline model if pattern of growth is complex. Disadvantages: Biased associations with outcome unless all random effects included. Pattern of growth not easy to interpret. Random effects may be highly correlated. More complex to fit than linear models.
Are there sub-groups of the population with different growth patterns? Do these groups have different outcomes?	As above.	*Growth Mixture Model* Advantages: Spline model more flexible than linear spline model, may be more realistic pattern of growth. Identifies latent sub-groups (all above methods assume there are no sub-groups). Disadvantages: Fairly complex to fit. Many parameters, so some assumptions need to be made. Pattern of growth may not be easily interpretable (graphs will be needed). Association is with group membership—can’t identify associations with specific periods of growth.

## Data accessibility

Due to ethical concerns, supporting data cannot be made openly available. The SWS team can provide the data on request subject to appropriate approvals. Researchers wishing to use the data would need to make a formal application to the SWS Oversight Group through the cohort PI (Hazel Inskip: hmi@mrc.soton.ac.uk) and ensure appropriate ethical approval is in place. Subject to approval and formal agreements being signed, the data would then be provided.
